# Psoriasis, Cardiovascular Events, and Biologics: Lights and Shadows

**DOI:** 10.3389/fimmu.2018.01668

**Published:** 2018-08-13

**Authors:** Giuseppina Caiazzo, Gabriella Fabbrocini, Roberta Di Caprio, Annunziata Raimondo, Emanuele Scala, Nicola Balato, Anna Balato

**Affiliations:** ^1^Department of Clinical Medicine and Surgery, University of Naples Federico II, Napoli, Italy; ^2^Department of Advanced Biomedical Sciences, University of Naples Federico II, Napoli, Italy

**Keywords:** anti-IL12/23, anti-IL17, anti-tumor necrosis factor-alpha, atherosclerosis, cardiovascular risk, psoriasis

## Abstract

Nowadays, it is well established a link between psoriasis and cardiovascular (CV) diseases. A series of different overlapping mechanisms including inflammation, homeostasis dysregulation, and genetic susceptibility are thought to underlie this association. Advances in understanding the molecular patterns involved in the complex scenario of psoriasis have highlighted a tight correlation with atherosclerosis. Indeed, common profiles are shared in term of inflammatory cytokines and cell types. In the last decade, the management of psoriasis patients has been revolutionized with the introduction of biological therapies, such as tumor necrosis factor-alpha (TNF-α), interleukin (IL)-12/23, and IL-17 inhibitors. In clinical setting, the effectiveness of these therapies as well as the incidence of CV events is related to the type of biologics. In particular, anti-TNF-α agents seem to reduce these events in psoriasis patients whereas anti-IL-12/23 agents related CV events reduction still remain to clarify. It has to be taken into account that IL-12/23 inhibitors have a shorter post-marketing surveillance period. An even more restricted observational time is available for anti-IL-17 agents. IL-17 is associated with psoriasis, vascular disease, and inflammation. However, IL-17 role in atherosclerosis is still debated, exerting both pro-atherogenic and anti-atherogenic effects depending on the specific context. In this review, we will discuss the differences between the onset of CV events in psoriasis patients, referred to specific biological therapy and the underlying immunological mechanism. Given the development of new therapeutic strategies, the investigation of these inhibitors impact on heart failure outcome is extremely important.

## Psoriasis and Cardiovascular (CV) Events

**Figure 1:**
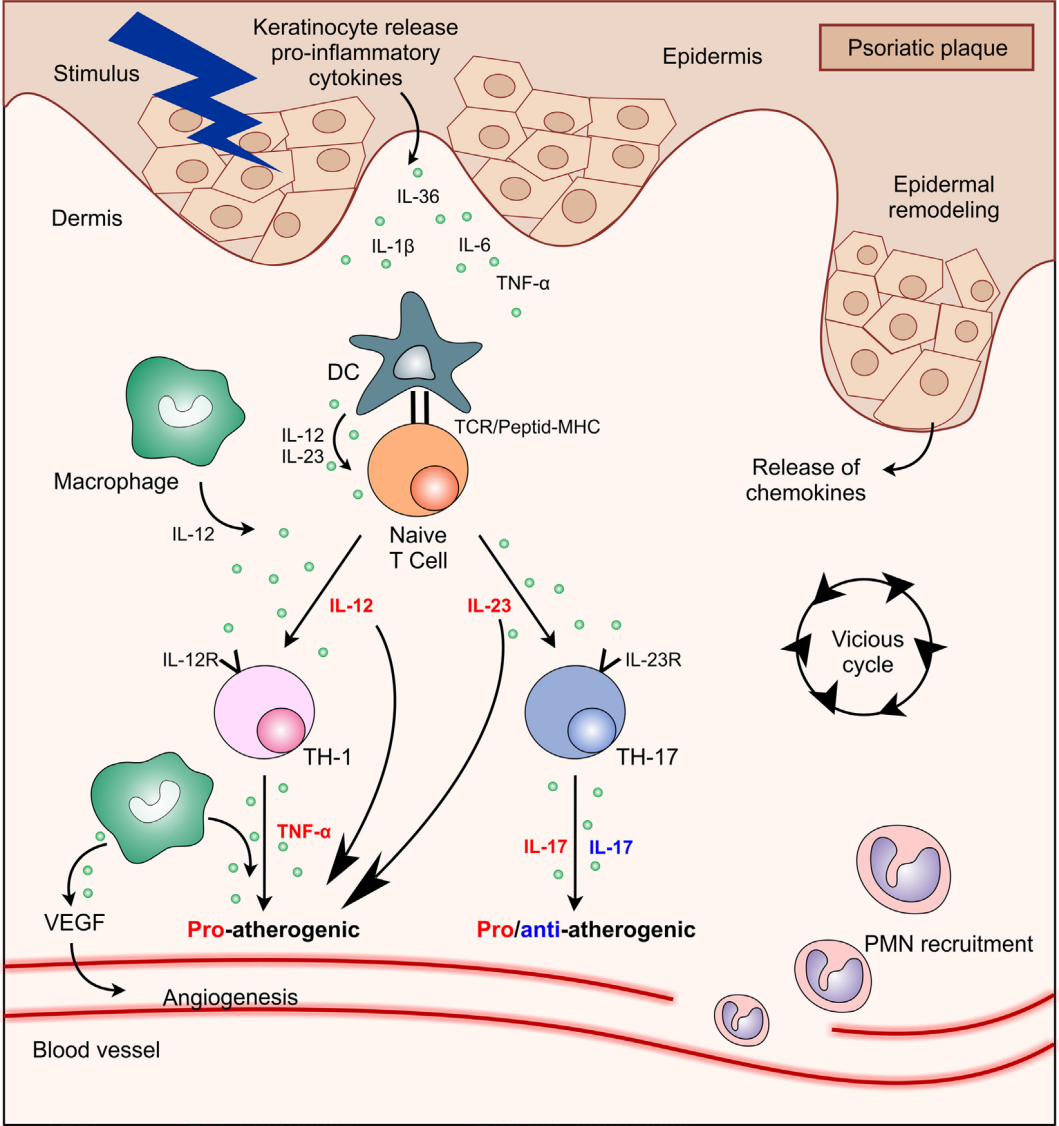
Interactions between main cell types and cytokines present in psoriasis plaque showing their functional significance in atherosclerosis process. Abbreviations: DC, dendritic cell; IL, interleukin; MHC, major histocompatibility complex; PMN, polymorphonuclear; TCR, T cell receptor; Th, T helper; TNF, tumor necrosis factor; VEGF, vascular endothelial growth factor.

The relationship between psoriasis (Pso) and an increased incidence of major adverse cardiovascular events (MACEs) has been observed for decades, since McDonald and Calabresi first demonstrated that the risk associated with arterial and vascular diseases was 2.2 times higher in more of 300 hospitalized patients with Pso compared with controls with other dermatological conditions (1). Since then, several studies have confirmed these findings, convincingly proving that patients with Pso have an effective higher risk of developing severe CV events, such as myocardial infarction (MI) and stroke (2). In 2006, using the General Practice Research Database (GPRD) source, Gelfand et al. suggested that Pso is an independent risk factor for acute MI and cardiovascular disease (CVD), particularly in young patients, and that this risk is most significant in patients with severe disease (3). In 2007, Ludwig and colleagues also identified Pso as a possible independent risk factor for CVD development founding a significantly increased prevalence and severity of the CVD indicator coronary artery calcification factor in these patients (4). In addition, increases in the prevalence of other independent “traditional” risk factors for CVD, including smoking, excess alcohol intake, as well as obesity, hypertension, dyslipidemia, and insulin resistance (the common underlying factors of metabolic syndrome), have been also reported in psoriatic patients (5–7). However, despite the evidences, some studies failed to find a significant independent association between Pso and CVD (8, 9). In 2015, using the same population-based GPRD, Parisi et al. conducted a series of multivariable analyses on patients with incident Pso concluding that neither Pso nor severe Pso are associated with a risk of MACE even after adjustment for traditional CVD risk factors (10). To date, the debate is whether or not this link represents a causal relationship or is a predisposition due to the underlying risk factors exhibited by patients with severe Pso (9, 10). The main hypothesis is that chronic inflammation which occurs in Pso is more than skin deep and results in a “psoriatic march” driving systemic mechanisms that are shared with other chronic inflammatory diseases, including atherosclerosis (Figure 1) (11–14). This concept was introduced for the first time in 2011 by Boehncke and colleagues to describe how systemic psoriatic inflammation may lead to insulin resistance as well as endothelial cell dysfunction, causing atherosclerosis, the major pathological change preceding MI and stroke development (15). Indeed, psoriatic patients with altered glucose metabolism and insulin resistance showed an increased arterial stiffness compared with healthy subjects, with a positive correlation between arterial stiffness and Pso disease duration (16, 17). Understanding why Pso may be a risk factor for atherosclerosis requires a basic understanding of their shared pathogenic features. In 2012, Flammer and Ruschitzka proposed the theory of “two plaques for one syndrome” since molecular mechanisms as well as pro-inflammatory cytokine profile of psoriatic lesions are remarkably similar to that of atherosclerosis ones, with a comparable inflammatory infiltrate of T cells, macrophages, and monocytes (18, 19). In addition, both diseases display a common pattern of T-cell activation, with T helper (Th)1 and Th17 cytokine upregulation, as well as increased local and systemic expression of adhesion molecules and endothelins (19). Thus, there are displacements of inflammatory cells between lesional psoriatic skin, peripheral circulation, and atheromatous plaques of coronary vasculature caused by the releasing cytokines derived from the skin and inflammatory mediators derived from Pso lesions into the circulation, together with an upregulation of cell adhesion molecules (20). Moreover, Pso-associated pro-inflammatory cytokines, such as interferon (IFN)-γ, tumor necrosis factor-alpha (TNF-α), and interleukin (IL)-17, have been found increased in atherosclerotic plaques and sera of patients with unstable CVD (21, 22). Similarly, increased expression of well known CV biomarkers, including monocyte chemoattractant protein (MCP)-1 and macrophage-derived chemokines, have been measured in the lesional skin and serum of psoriatic patients suggesting shared inflammatory pathways linking Pso and CVD (23). Recently, Kolliker Frers et al. have demonstrated that pro-atherogenic inflammation marker C-reactive protein (CRP) and soluble intercellular adhesion molecule-1 levels are increased also in psoriatic patients with no CV history or traditional CV risk factors, compared with healthy subjects, as well as in patients with recent-onset PsA, even in the absence of CV risks. These data reinforce the concept that that the degree of atherosclerosis tendency might be related to the amount of the inflammatory psoriatic burden and highlight the importance of primary prevention in Pso also in those psoriatic patients with no history of CV events (24).

## Therapeutic Implications

The growing body of evidences pointing to increases in MI and CV events in psoriatic patients has raised the question whether treating cutaneous disease might also prevent heart attacks and decrease CVD risk development. Moreover, the postulated hypothesis that inflammatory cascade activated in Pso contributes to the atherosclerotic process has laid the groundwork for supposing that the anti-inflammatory Pso therapy could theoretically improve also atherosclerosis and reduce the risk of MACE (25). Prodanovich et al. first reported that treatment with methotrexate (MTX), especially when used at low doses and in combination with folic acid supplementation, was able to reduce the rate of vascular disease in patients with Pso or rheumatoid arthritis (RA) (26). A subsequent meta-analysis of 10 studies confirmed these data concluding that the use of MTX resulted in 21% lower risk of CVD and 18% lower risk of MI (27). Moreover, data from a large health-care system in USA proved that decreased MI risk in psoriatic patients treated with MTX was greater than those receiving topical therapy (28). In 2013, using a population-based Danish cohort, an observational study found that the risk of MACE development decreased in psoriatic patients in treatment with MTX compared with other non-biological agents, including oral retinoids, cyclosporine, and phototherapy (29). Indeed, treatment with cyclosporine, leading to impaired renal function and hypertension, may negatively affect the MACE profile in these patients by increasing the risk of hypertension and dyslipidemia (30). For that reason, in psoriatic patients, cyclosporine should only be used for a limited period and should be substituted with another systemic agent once the skin condition is improved (20). Similarly, acitretin, the most commonly agent used to treat generalized pustular Pso, it has been associated with an increased risk of hypertension, hyperlipidemia, and CVD even if several investigations have shown that retinoids also improve and ameliorate the formation of atherosclerotic plaques (31, 32). Moreover, Boehncke et al. found that also treatment with fumaric acid esters resulted in an improvement of CV risk biomarkers in patients with moderate-to-severe plaque-type Pso. In particular, the authors demonstrated that a continuous systemic therapy with fumaric acid esters, behind reducing Pso severity, was able to improve the endothelial vasodilator function, reduce serum levels of CRP, and increase the potentially cardioprotective adiponectin (33). However, it has been with the introduction of biological therapies that Pso treatment expectations and long-term control have greatly improved and the idea that these therapies, more than the systemic ones, might reduce the risk of CVD reinforced (34). To date, although several biological therapies have been approved and licensed for the treatment of Pso, their CV safety profile is not yet well established (35). Currently, it is unclear whether any of these therapies, which include TNF-α inhibitors (TNFi) (infliximab, etanercept, and adalimumab); inhibitors of the p40 subunit common to IL-12 and IL-23 (ustekinumab and briakinumab); IL-17A inhibitors (secukinumab and ixekizumab), and its receptor antagonist (brodalumab) could alter the risk of CVD development (35). Thus, the aim of this review has been to evaluate whether possible associations exist between currently licensed biological therapies and risk of MACEs in adult patients with Pso.

## Psoriasis as Immune-Mediated Disease

Psoriasis is an immune-mediated inflammatory skin disorder that affects 2.5% of the population worldwide. While the exact etiology of psoriasis is unknown, genetic and environmental factors are important in disease development (36, 37). Moreover, the immune system plays a crucial role in the overall disease pathogenesis, with various innate and adaptive immune cells and pro-inflammatory mediators involved at different stages of the disease (38). T cells are known to be the main actors in the pathogenesis, in particular Th1 and Th17 lymphocytes contribute through the inflammatory cytokines release that promote further recruitment of immune cells, keratinocyte proliferation, and inflammation (39). More specifically, CD4+ and CD8+ T cells with an IL-17-secretory phenotype are important contributors owing to their production of the pro-inflammatory cytokines IL-17, IL-22, and TNFα (40). For over 30 years, Pso was thought to involve a Th1 response, driven by the cytokines IFN-γ and IL-12; however, the discovery of the Th17 cell population has revolutionized the complex scenario of Pso. Indeed, the IL-23/Th17 cell signaling axis effects on keratinocytes and infiltrating immune cells in the skin has shaped the current disease model of Pso. Therefore, psoriatic disease is understood as a patterned response to chronic activation of the IL-23/Th17 axis (36). Recently, it has been identified T-regulatory cells expressing IL-17A in which, Foxp3 expression is progressively lost, whereas RORγ expression is increased (39, 40). This process is upregulated by IL-23 and concurring to the chronic inflammation seen in Pso. In particular, IL-23 regulates the maintenance of Th17 cells, whereas IL-17 and TNF mediate effector functions of innate (TNF) and adaptive (TNF, IL-17) immune cells.

## Effects of Biological Therapy on CV Risk in Psoriatic Patients

### Anti-TNF-α Agents

Pso is considered a T cell-mediated immune disease with a mixed Th1/Th17 cytokines environment. The interplay of these cytokines has a central role in the disease process by causing the spread of local inflammation at systemic levels. Specifically, elevated levels of TNF-α and soluble TNF receptors have been found in lesional psoriatic skin and in the serum of patients with severe Pso, which similarly reflect those detected in congestive heart failure (CHF) patients (41–43). TNF-α activation system can lead to different outcomes: (i) the development of atherosclerosis, (ii) the deterioration of cardiac functions, and (iii) the vascular smooth muscle cell remodeling (44–46). Since 2004, US Food and Drug Administration (FDA) has approved TNFi, such as etanercept, infliximab, and adalimumab for Pso treatment. Most recently, five TNFi biosimilars have been approved by FDA for Pso: infliximab-dyyb, infliximab-abda, etanercept-szzs, adalimumab-atto, and adalimumab-adbm (47, 48). The effectiveness of these biologics in the management of psoriatic patients has been highlighted since the earliest literature (49–54). Anti-TNF-α agents can reduce the CRP, the vascular endothelial growth factor (VEGF), and the chemotactic factors (e.g., VCAM-1, E-selectin, IL-8, and MCP-1) (55, 56) as well as the Th17 cell count in the peripheral blood of psoriatic patients (49, 57). In fact, TNFi stop CD4+ T cells differentiation into Th1, Th17, and Th22 cells and the consequent release of IL-17A, IL-17F, and IL-22 (47, 58). Although the evidence indicate that TNFi are effective in reducing the systemic inflammation, it is still debated whether anti-TNF-α agents can decrease CV risk in these patients or they only represent a random association. It has been reported in literature that psoriatic patients who received TNFi showed an improvement of psoriasis area severity index (PASI) score characterized by a reduction of CV risk biomarkers (e.g., CRP, VEGF, and resistin serum levels) after 24 weeks of therapy (59). Additional studies have highlighted that the vascular function was somehow restored after 10 weeks of etanercept treatment, leading to a significant reduction in CRP levels and improvements of insulin sensitivity (60, 61). The same authors also proved that TNFi statistically led to a significant reduction (*P* = 0.0001) of retinol-binding protein 4 (RBP4) circulating levels in psoriatic patients (62). RBP4 is highly linked to subclinical atherosclerosis, due to its positive correlation to carotid intima-media thickness (IMT) indicator (61, 62). Recently, it has been assumed that psoriatic patients treated with TNFi showed IMT indicator decrease associated with appropriate therapeutic responses (63). Another experimental study has reported the aortic inflammation reduction by ^18^F-fluoro-deoxyglucose (FDG)-positron emission tomography/computed tomography (CT) in 30 psoriatic patients who received adalimumab (64). Similarly, the TNFi effects on the development of atherosclerosis has been studied in 58 psoriatic patients during a 13-month period by CT imaging (65). In addition, the patients who did not received TNFi showed a significant progression of the coronary calcification, while the TNFi-treated group did not. Another research based on echocardiographic data confirmed the improvement of the myocardial function in 18 psoriatic subjects treated with TNFi and IL12/23 inhibitors (66). Likewise, right ventricular systolic function was improved in a 30-month study of 44 psoriatic patients treated with TNFi (67). Therefore, anti-TNF-α agents might be identified as CV protective factors together with statin drug, female gender, and age (68). However, no therapeutic effect of TNFi has been demonstrated in CHF patients classified as grade III–IV by the New York Heart Association. Initially, etanercept seemed to have beneficial effects in heart failure patients (69). Subsequently, a large study consisting of 1,500 patients with symptomatic heart failure has shown no relation to mortality or hospitalizations after treatment with etanercept (70). Other clinical trials have reported a higher percentage of mortality in CHF patients who received infliximab at 10 mg/kg (higher dose than that used in Pso and RA) than the controls (71, 72). These preliminary discovers have provided the evidence for the contraindication against the use of TNFi in CHF patients. Nowadays, anti-TNF-α drugs are not contraindicated in patients with heart diseases other than CHF (34). Random control trials (RCTs) including severe psoriatic patients who received at least one of TNFi, resulted in a number of MACEs (35, 73–76). Overall, the pooled analysis confirmed that no statistically significant difference exists between patients treated with biologics or placebo (35) (Table 1). Considering TNFi separately, there was also no statistically significant difference in patients receiving etanercept, infliximab, or adalimumab (35). In addition, the incidence rates (IRs) of MACEs were reduced by the use of anti-TNF-α agents respect to conventional therapies (28, 29, 77, 78). The use of TNFi for patients with Pso or psoriatic arthritis was associated with a 55% reduction in the incidence of MI compared with those patients who were only treated with topical therapy (28). Similarly, psoriatic patients treated with TNFi displayed a significantly lower risk of MI when compared with MTX (79). Furthermore, treatment with TNFi is associated with an 11% reduced CV risk every 6 months of additional treatment (79). On the other hand, some authors suggested that these therapies do not seem to reduce the risk of MI, because they increase patients’ weight and cholesterol levels after 3 or 6 months of therapy (66, 79, 80). These results could be related to a subsequently biological effect of TNFi due to neutralization of TNF-α cachetic properties (81). On the contrary, stable lipids levels were found in psoriatic patients treated with adalimumab, etanercept, or infliximab for 13 months (65). Psoriatic patients randomized to adalimumab or non-systemic treatment (topical therapies or phototherapy) showed no changes in total cholesterol, high-density lipoprotein, low-density lipoprotein, or triglycerides after 15 weeks of treatment (64). Despite some scientific reports overshadows TNFi role on CV risk, current studies support TNFi benefic effects on cardio metabolic parameters. To conclude, although literature remains heterogeneous, in part due to methodological differences, anti-TNF-α agents exert a protective effect on CV risk.

**Table 1:** Main randomized controlled trials and meta-analysis studies on the rates of major adverse cardiovascular events (MACEs) in psoriatic patients treated with biological agents.

Biological agents	Reference	No. of patients or trials	MACE risk (IR or OR)	Follow-up period
TNFi	Rungapiromnan et al. (35)	18 RCTs comparing TNFi (4 adalimumab, 9 etanercept, 5 infliximab) vs placebo	OR, 0.67 (95% CI, 0.10–4.63, *P* = 0.69)	8–50 weeks

Ustekinumab (anti-IL12/23p40)	Reich et al. (82, 83)	1582 ustekinumab vs 732 placebo-treated patients	IR, 0.3%; (95% CI, 0.1–0.70) vs 0.0% (95% CI, 0.0–0.5%)	20 weeks
	Tzellos et al. (84)	9 RCTs (ustekinumab vs placebo)	OR, 3.96 (95% CI, 0.51–30.41; *P* = 0.19)	30 weeks
	Rungapiromnan et al. (35)	7 RCTs (ustekinumab vs placebo)	OR, 4.48 (95% CI, 0.24–84.77; *P* = 0.32)	30 weeks

Briakinumab (anti-IL12/23p40)	Langley et al. (85)	1258 briakinumab vs 624 placebo-treated patients	IR, 1.33% (95% CI, 0.43–3.10) vs 0.60% (95% CI, 0.35–0.94)	12 weeks
	Tzellos et al. (86)	9 RCTs (briakinumab vs placebo)	OR, 4.47 (95% CI, 0.69–28.89; *P* = 0.12)	30 weeks

Ustekinumab and Briakinumab	Tzellos et al. (86)	9 RCTs (ustekinumab and briakinumab vs placebo)	OR, 4.23, (95% CI, 1.07–16.75; *P* = 0.04)	30 weeks

Secukinumab (anti-IL17)	van de Kerkhof et al. (87)	10 phase II/III clinical trials	IR, 0.35% (95% CI, 0.10–0.90)	52 weeks
	Egeberg et al. (88)	196 patients	IR, 3.1% (95% CI, 1.1–8.1)	104 weeks

Ixekizumab (anti-IL-17)	Strober et al. (89)	7 RCTs (UNCOVER-1, -2, and -3 plus an additional 4 phase I–III studies)	38 [0.6]^a^	60 weeks

Brodalumab (anti-IL17R)	Papp et al. (90)	1 phase III clinical trial (AMAGINE-1)	5 (1.0)^b^	52 weeks

*IR, incidence rate; OR, odds ratio; CI, confidence interval; RCTs, randomized controlled trials*.

*^a^MACE occurring in ≥1 patient, n [IR]*.

*^b^n (exposure-adjusted event rate per 100 patient-years)*.

### Anti-IL-12/23 Agents

The basis for developing anti-IL-12/23 biological drugs was encouraged by evidences that mice deficient in the subunit p40, shared by both IL-12 and IL-23, are resistant to experimentally induced autoimmune conditions, such as psoriasis (91, 92). Levels of IL-12/23p40 mRNA are more elevated in psoriatic than in healthy skin (93, 94). Cytokines induced by IL-12 (such as IFN-γ) and by IL-23 (such as IL-17A, IL-17F, and IL-22) are increased in psoriatic plaques (94). Moreover, in the last two decades, these inflammatory mediators have also been shown to contribute in CVD development. Indeed, it has been shown that serum levels of IL-12 and IL-23 are augmented in patients with CVD (95, 96). IL-12 and IL-23 are presented in atherosclerotic plaque and thereby affecting the pro-inflammatory status in these patients (96–98). Taken together, these evidences suggest that targeting IL-12/23 could represent a valid therapeutic option for psoriatic disease, with benefits on cutaneous involvement and on CV comorbidities. The efficacy of anti-IL-12/23p40 biological drugs (ustekinumab and briakinumab) for psoriasis treatment (Table 2) has been evaluated in two phase III RCTs and in four phase III RCTs, respectively (82, 85, 99–103). The hypothesis that these agents could possibly increase CV risk while improving cutaneous disease is unexpected. Nevertheless, safety concerns have been raised regarding the possibility of an increased risk of MACEs with the utilize of anti-IL-12/23 biological agents. We will discuss on lights and shadows of this hot question. In 2011, Reich et al. (83) evaluated this point due to the fact that in the ustekinumab clinical studies target population reported a higher rate of MACEs than placebo one. In the 1,582 ustekinumab-treated patients enrolled in the phase II and III placebo-controlled psoriasis studies, five MACE events occurred [0.3%; 95% confidence interval (CI), 0.1–0.7%] respect to no events in 732 placebo-treated patients (0.0%; 95% CI, 0.0–0.5%) (83). In particular, an important numerical imbalance in MACE rate was observed in the phase II trial with a 4:1 randomization ratio (risk difference 1.2%; 95% CI, 3.9–3.7%). However, the two phase III trials did not replicate a same high risk difference (0.2%; 95% CI, 1.2–1.2 and 0.1%; 95% CI, 0.7–0.7% for PHOENIX 1 and 2, respectively) (83) (Table 1). During the 12/20-week follow-up periods, MACE events did not show tendency to cluster. Indeed, the five MACEs occurred at weeks 2, 6, 10, 14, and 17 (83). The standardized IR in these studies ranged between 0.34 and 0.52, a rate lower than that estimated for the general population in the USA or in psoriatic patients (83). Moreover, all patients who experienced MACEs had at least three established CV risk factors. Longer term analyses demonstrated that rates of CV events remained low with up to 3 years. However, the absence of a control group precludes definitive assessment of the effect of ustekinumab on MACE risk (83). Thus, data from short-term, controlled trials give only partial information regarding the possible impact of ustekinumab on CV risk: the results suggest neither harmful nor beneficial effects. However, a smaller risk increase cannot be totally ruled out. Concerning another anti-IL-12/23p40 monoclonal antibody, briakinumab, the question is even more burning. Results from one of the four clinical trials have reported more MACE events in briakinumab-treated patients compared with placebo ones over a 12-week period (101). In details, 18 MACEs had been recorded with 4 CV deaths compared with no events in placebo group. The frequency of MACE was spread in an apparently uniform manner over time. The standardized IRs were 1.33/100 patient-years (95% CI, 0.43–3.10) respect to 0.60 (95% CI, 0.35–0.94) in the placebo-controlled phase (Table 1). In patients with two or more CV risk factors, the IR was more elevated (2.15 events/100 patient-years). For all these reasons, the study protocol underwent an amendment (85). Interestingly, no MACEs were reported during other two published clinical trials (briakinumab vs placebo or etanercept) as well as in another trial in which briakinumab was compared with MTX with a 52-week follow-up (82, 102, 103). However, the application for authorization to market briakinumab has been withdrawn.^1^ Overall, definitive conclusions did not could be performed. For this reason, two industry-independent meta-analyses of nine randomized, double-blind, placebo-controlled, monotherapy trials have been conducted to deeper examine the possible association of MACEs with anti-IL-12/23 agents (84, 104) (Table 1). The meta-analysis by Ryan et al. found no increased risk of MACEs in treated patients compared with placebo ones, using the Mantel–Haenszel fixed-statistical effects model (104). On the other hand, Tzellos et al. analysis showed that the odds ratio (OR) for briakinumab- and ustekinumab-treated patients was not statistically significant (OR 4.47, 95% CI, 0.69–28.89, *P* = 0.12; OR 3.96, 95% CI, 0.51–30.41, *P* = 0.19, respectively). When combined, the OR for MACEs between patients treated with biological agents and those receiving placebo was found to be statistically significant (OR = 4.23, 95% CI, 1.07–16.75, *P* = 0.04) (84). In this case, the statistical effects model used was the Peto One-Step OR method. The divergence in these results has been attributable to the use of two different methods to estimate the risk. The Mantel–Haenszel fixed-statistical model using zero-cell corrections provides estimates for all studies, including zero event ones. This make it less suitable for meta-analysis of rare events (105–108) resulting in low statistical power. MACE event rates were estimated to be 0.28, 0.35, and 0.31% for ustekinumab, briakinumab, and both, respectively (108). It has been established that in case of an event IR of 1% or less, the best statistical approach is Peto method (105). However, the Peto OR method excluding trials with zero events from the analysis can lead to an overestimation of true relative risk. Moreover, neither the analysis by Tzellos et al. nor that by Ryan et al. have been adjusted for dropouts; this likely resulted in an overestimation of true risk (106). A large 5-year post-marketing study on the use of ustekinumab for psoriasis did not support the hypothesis of an augmented risk of MACEs (109). Moreover, an imbalance in MACE event rate has not been reported in other therapeutic indications of ustekinumab, such as psoriatic arthritis and inflammatory bowel disease (110–112). In addition, to date, the FDA has not communicated any changes regarding the prescribing for ustekinumab related to CV risk. More recently, a new meta-analysis has been performed by Rungapiromnan et al. (35) to evaluate the impact of biological therapies on risk of MACEs (Table 1). In this meta-analysis, they have selected RCTs in which patients received only licensed dose regimens of biological therapies. For this reason, briakinumab has been excluded from the analysis. Peto ORs with 95% CIs has been applied for statistical analysis. Regarding ustekinumab, no statistically significant difference in risk of MACEs has been reported respect to placebo (OR 4.48, 95% CI, 0.24–84.77, *P* = 0.32); similarly, comparing ustekinumab 45 mg with 90 mg the OR was not statistically significant (OR 1.00, 95% CI, 0.06–16.03, *P* = 1.00) (35). The most important limitation is that these findings were mainly based on small sample sizes and short-term follow-up (ranging from 12 to 30 weeks). This is an important aspect to considering since it has been demonstrated that during the initial stage of therapy with ustekinumab inflammatory pro-atherogenic mediators such as IL-12/23 p40 temporarily increased and then dramatically decreased at week 32 (113, 114). Thus, other post-marketing studies and novel larger randomized controlled trials will be needed to continue the surveillance to assess the potential association connecting the use of anti-IL-12/23p40 biological drugs and increased CV risk.

**Table 2:** FDA approval biological drugs for psoriasis.

Biological drug	Biological structure	Mechanism of action	FDA approval for psoriasis (year)
Etanercept	Soluble TNFR2 coupled to Fc portion of IgG1	Anti-TNF-α	2004
Infliximab	Human/mouse chimeric IgG1 mAb	Anti-TNF-α	2006
Adalimumab	Human IgG1 mAb	Anti-TNF-α	2008
Ustekinumab	Human IgG1 mAb	Anti-p40 IL-12/23	2009
Secukinumab	Human IgG1κ mAb	Anti-IL17A	2015
Adalimumab-atto (biosimilar)	Human IgG1 mAb	Anti-TNF-α	2016
Etanercept-szzs (biosimilar)	Soluble TNFR2 coupled to Fc portion of IgG1	Anti-TNF-α	2016
Infliximab-dyyb (biosimilar)	Human/mouse chimeric IgG1 mAb	Anti-TNF-α	2016
Ixekizumab	Humanized IgG4 mAb	Anti-IL17A	2016
Adalimumab-adbm (biosimilar)	Human IgG1 mAb	Anti-TNF-α	2017
Infliximab-abda (biosimilar)	Human/mouse chimeric IgG1 mAb	Anti-TNF-α	2017
Brodalumab	Human IgG2 mAb	Anti-IL17RA	2017
Guselkumab	Human IgG1λ mAb	Anti-p19 IL-23	2017

*IgG, immunoglobulin G; IL, interleukin; mAbs, monoclonal antibodies; IL-17RA, interleukin receptor A; p40, subunit 40; p19, subunit 19; TNFR2, tumor necrosis factor receptor 2; TNF-α, tumor necrosis factor; FDA, Food and Drug Administration*.

### Anti-IL17 Agents

In the past decade, the research has been focused on Th17 cells, which during differentiation secrete IL-17. IL-17 cytokine family consists of six members (IL-17A, B, C, D, E, and F). IL-17A (referred to here as IL-17) is among them, the major isoform (115). IL-17 is produced by Th17 cells and also by other cell subtypes, including γδ T, natural killer, etc. (116). However, interest in the Th17 population in relationship to Pso increased when numbers of circulating Th17 cells and IL-17 expression levels were found upregulated in the Pso lesional skin compared with non-lesional skin (49, 117). Recently, several anti-IL-17 agents have been developed. These primarily include anti-IL-17A monoclonal antibodies, secukinumab and ixekizumab, and brodalumab, an anti-IL-17 receptor monoclonal antibody. These biological agents that target the IL-17 signaling pathway have currently been evaluated and approved for the treatment of moderate-to-severe plaque Pso. The results reported by studies on efficacy and safety of IL-17 inhibitors are very promising. They showed the superiority of these new biological agents respect to both placebo and other biologics, such as ustekinumab and etanercept. For the first time, these trials include as end point the percentage of patient that achieving 90 or 100% of clinical improvement (PASI-90 or PASI-100, respectively) (47). In addition, although IL-17 antagonists had a higher rate of any adverse events than placebo, there was no significant difference in severe adverse events. This suggests that IL-17 antagonists are well tolerated (118). Even if the effects of IL-17 therapy on CVD in psoriatic patients is yet to be fully elucidated. Indeed, the interest on MACE is a crucial and much debated point. Likewise, Th17 pathway has a crucial role in CVD (22). Indeed, IL-17 is also involved in angiogenesis process, and on the synthesis of MMPs and CRP. Thus, in theory, Th17 cells could also play a crucial role in atherosclerotic and in CVD. Reports in atherosclerosis showed a conflicting result on IL-17 and Th17 cells in disease onset and plaque stability. IL-17 may exhibit both pro- and anti-atherogenic effects, depending on the inflammatory context (115). IL-17 pro-atherogenic effects may result from the induction of pro-inflammatory cytokines or chemokines (IL-6, GM-CSF, CCL2, and CXCL1) by endothelial cells or macrophages. The IL-17 atheroprotective effects may be dued by IFN-γ decreased and to its inhibitory effect on the expression of vascular cell adhesion molecule, this molecule is important to mediating the accumulation of monocytes and T cells within the lesions. Indeed, it was showed that IL-17, IL-21, and IL-23 were found in atherosclerotic plaques and associated with increased inflammation and plaque vulnerability (119). By contrast, Taleb et al. have reported a role for IL-17 in promoting plaque stability. Indeed, IL-17 expression in human carotid lesions was related to a fibrous phenotype with a lower macrophage and higher smooth muscle cell content (120). Despite some data suggesting increased levels of Th17 cells and IL-17 in patients with acute coronary syndromes (121, 122), the bulk of evidence indicate that circulating IL-17 levels are similar in patients with or without coronary artery disease (123). Indeed, some authors have reported that the patients with IL-17 low levels were more susceptible to the risk of death and recurrent MI (124). On the other hand, several experimental evidences and biomarker studies, which indicate a link between IL17 and instability in atherosclerotic plaques, supporting the hypothesis that in part explain the high risk of MI in Pso patients (22). Indeed, Pso patients have significantly elevated IL-17 serum levels (49) and they have a high risk to developing CV comorbidities. The conception that Th17 cytokines may provide a link between Pso and CVD comes from literature data that reporting a pathogenic role of IL-17 in Pso and examining the Th17 axis contribution into atherosclerosis (125).

As regarding secukinumab, data reported that there is a favorable safety profile in patients with moderate-to-severe plaque Pso over a total follow-up period of 52 weeks in a pooled safety analysis of 10 studies. However, at baseline, more subjects in the secukinumab groups had CV risk than comparator groups. Even if overall exposure-adjusted IRs of adjudicated MACE in secukinumab-treated subjects was comparable to etanercept-treated subjects (87). During the first 12 weeks of secukinumab studies, MACEs were reported in three patients receiving secukinumab 300 mg (0.26%), in no patients receiving etanercept, and in one patient treated with placebo. Over 52 weeks, exposure-adjusted IRs of MACEs were comparable in patients receiving secukinumab 300 mg (0.42/100 subject-years), secukinumab 150 mg (0.35/100 subject-years), and etanercept (0.34/100 subject-years), despite the presence of higher baseline CV risk factors in the two secukinumab groups (Table 1). All patients with a MACE had CVD risks, such as hypertension, dyslipidemia, etc. (87). In summary, from this comprehensive analysis of pooled safety data from 10 studies reported that the incidences of MACE in secukinumab-treated patients was low. A very recent real-life observational study of 195 patients (Table 1) treated with secukinumab for up to 2 years reported that 2% of patients suffered a CV event yielding a conspicuously elevated IR compared with findings from the secukinumab phase III clinical trial program, although the absolute numbers were very low (*n* = 4) (88).

The other antibody that inhibits IL-17 is ixekizumab; recently, safety data from a 12-week induction period, a 12- to 60-week maintenance period, and from all ixekizumab-treated patients were presented. The analysis of these data essentially reported similar rates of MACE between ixekizumab and etanercept and low rates of MACE with continued exposure for ixekizumab until week 60 (Table 1) (89). In particular, during the induction period, the between-group rates of adjudicated MACE were similar. The MACE individual components did not change substantially with longer exposure to ixekizumab. 7 of 4,035 ixekizumab-treated patients experienced vascular death. At baseline, patients subsequently developing MACE had a higher prevalence of risk factors for acute atherothrombotic events than patients without MACE. In conclusion, ixekizumab use was not associated with an increased risk of MACE. The safety profile reported in this analysis study is consistent with previous reports for ixekizumab (76, 126).

As regarding Brodalumab that selectively targets human IL-17RA and antagonizes the IL-17 pathway, it was considered safety from analysis of a prospective study, indeed incidences of serious adverse events in the induction phase were low among groups. There were no reports of MACEs during the induction phase, and only five reports (exposure-adjusted event rate of 1.0/100 patient-years) through week 52 (Table 1) (90). Conversely, in another phase III study, brodalumab treatment was compared with ustekinumab treatment in psoriasis, and the adverse effects were more recurrent in the brodalumab group than in the ustekinumab one. Indeed, in the AMAGINE-2 study, one death by stroke happened during the induction phase, and five deaths occurred through week 52; whereas in the AMAGINE-3 study, two deaths occurred: one from cardiac arrest and one from accidental death (127). However, the sizes of study populations, which were adequate for efficacy and common adverse event assessments, may have been inadequate to assess rare adverse events, which would require longer follow-up of large numbers of patients to better understand the safety profile of brodalumab.

Taken together, results from short-term safety and efficacy trials exploring anti-IL-17 therapy in psoriatic patients suggest no increased CVD risk compared with placebo or other classes of biologics used in Pso. Indeed, we may infer that IL-17 antagonists could possibly play an outstanding role in reducing CV morbidity. Even if there are several limitations in these clinical trials, e.g., patients highly selected, short duration of treatment with placebo or TNFi in relative few number of patients compared with the number of patients receiving IL-17 inhibitors. The advent of IL-17 biologics has improved significantly Pso disease (Table 2), but time will tell whether this IL-17 inhibition will also improve CVD outcomes in Pso.

## Conclusion

Since systemic inflammation is currently considered the main cause of CVD risk in Pso, it has been thought that biological therapies might be helpful in treating not only cutaneous manifestations but have also positive or negative CV effects depending on the specific cytokine target and molecular mode of action. However, despite the recognized efficacy of biological agents in psoriasis treatment, little is known about their impact on risk of MACE in these patients. Data from meta-analyses have suggested that biological therapies including TNFi, anti-IL-12/23p40, and IL-17A agents did not have a significant impact on the risk of MACE in psoriatic patients over the short term. In particular, the use of TNFi is currently associated with a minor CVD risk in these patients, whereas the role of anti-IL-12/23p40 agents remains conflicting. Nevertheless ustekinumab, the only IL-12/23p40 inhibitor currently approved for Pso, results neutral on CV parameters. Regarding newer IL-17 inhibitors, early data have suggested no increased CVD risk respect to placebo or other classes of biologics used in psoriasis. However, because of follow-up short duration, many of these results should be interpreted with caution. Moreover, more studies, involving recruitment of a larger numbers of patients as well as a longer duration of treatment exposure, are needed to better evaluate the impact of biological therapies on the risk of MACEs in patients with Pso and to develop effective strategies for CVD prevention in these patients.

## Author Contributions

AB designed the manuscript structure, reviewed the scientific literature, and supervised the final version of the manuscript. GC designed the review structure and contributed to the writing of the manuscript. ES contributed to the writing of the manuscript and created graphical illustration. RC and AR reviewed the scientific literature and contributed to the writing of the manuscript. GF and NB reviewed the scientific literature and approved the final version of the manuscript.

## Conflict of Interest Statement

The authors declare that the research was conducted in the absence of any commercial or financial relationships that could be construed as a potential conflict of interest.

## Footnotes

^1^Available from: http://www.ema.europa.eu/docs/en_GB/document_library/Press_release/2011/01/WC500100769.pdf (Accessed: Febraury 8, 2018).

## Abbreviations

CI, confidence interval; CRP, C-reactive protein; CT, computed tomography; CV, cardiovascular; CVD, cardiovascular disease; FDG, ^18^F-fluoro-deoxyglucose; GPRD, General Practice Research Database; HDL, high-density lipoprotein; IFN, interferon; IL, interleukin; IMT, intima-media thickness; IRs, incidence rates; LDL, low-density lipoprotein; MACEs, major adverse cardiovascular events; MCP, monocyte chemoattractant protein; MI, myocardial infarction; MTX, methotrexate; OR, odds ratio; PASI, psoriasis area severity index; PET, positron emission tomography; RBP4, retinol-binding protein 4; RCTs, random control trials; Th, T helper; TNF, tumor necrosis factor; TNFi, TNF-α inhibitors; V-CAM, vascular cell adhesion molecule; VEGF, vascular endothelial growth factor.

## References

[1] McDonald CJ Calabresi P Occlusive vascular disease in psoriatic patients. N Engl J Med (1973) 288:912 10.1056/NEJM197304262881715 4692910

[2] Samarasekera EJ Neilson JM Warren RB Parnham J Smith CH. Incidence of cardiovascular disease in individuals with psoriasis: a systematic review and meta-analysis. J Invest Dermatol (2013) 133:2340–6. 10.1038/jid.2013.149 23528816

[3] Gelfand JM Neimann AL Shin DB Wang X Margolis DJ Troxel AB. Risk of myocardial infarction in patients with psoriasis. JAMA (2006) 296:1735–41. 10.1001/jama.296.14.1735 17032986

[4] Ludwig RJ Herzog C Rostock A Ochsendorf FR Zollner TM Thaci D Psoriasis: a possible risk factor for development of coronary artery calcification. Br J Dermatol (2007) 156:271–6. 10.1111/j.1365-2133.2006.07562.x 17223866

[5] Naldi L Chatenoud L Linder D Belloni Fortina A Peserico A Virgili AR Cigarette smoking, body mass index, and stressful life events as risk factors for psoriasis: results from an Italian case-control study. J Invest Dermatol (2005) 125:61–7. 10.1111/j.0022-202X.2005.23681.x 15982303

[6] Sommer DM Jenisch S Suchan M Christophers E Weichenthal M. Increased prevalence of the metabolic syndrome in patients with moderate to severe psoriasis. Arch Dermatol Res (2006) 298:321–8. 10.1007/s00403-006-0703-z 17021763

[7] Padhi T Garima. Metabolic syndrome and skin: psoriasis and beyond. Indian J Dermatol (2013) 58:299–305. 10.4103/0019-5154.113950 23919003 PMC3726879

[8] Wakkee M Herings RM Nijsten T Psoriasis may not be an independent risk factor for acute ischemic heart disease hospitalizations: results of a large population-based Dutch cohort. J Invest Dermatol (2010) 130:962–7. 10.1038/jid.2009.321 19812600

[9] Stern RS. Psoriasis is not a useful independent risk factor for cardiovascular disease. J Invest Dermatol (2010) 130:917–9. 10.1038/jid.2009.446 20231828

[10] Parisi R Rutter MK Lunt M Young HS Symmons DPM Griffiths CEM Psoriasis and the risk of major cardiovascular events: cohort study using the clinical practice research datalink. J Invest Dermatol (2015) 135:2189–97. 10.1038/jid.2015.87 25742120

[11] Eder L Gladman DD. Atherosclerosis in psoriatic disease: latest evidence and clinical implications. Ther Adv Musculoskelet Dis (2015) 7:187–95. 10.1177/1759720X15591801 26425147 PMC4572363

[12] Chiricozzi A Raimondo A Lembo S Fausti F Dini V Costanzo A Crosstalk between skin inflammation and adipose tissue-derived products: pathogenic evidence linking psoriasis to increased adiposity. Expert Rev Clin Immunol (2016) 12:1299–308. 10.1080/1744666X.2016.1201423 27322922

[13] Raimondo A Lembo S Di Caprio R Donnarumma G Monfrecola G Balato N Psoriatic cutaneous inflammation promotes human monocyte differentiation into active osteoclasts, facilitating bone damage. Eur J Immunol (2017) 47:1062–74. 10.1002/eji.201646774 28386999

[14] Boehncke WH Systemic inflammation and cardiovascular comorbidity in psoriasis patients: causes and consequences. Front Immunol (2018) 9:579 10.3389/fimmu.2018.00579 29675020 PMC5895645

[15] Boehncke WH Boehncke S Tobin AM Kirby B. The ‘psoriatic march’: a concept of how severe psoriasis may drive cardiovascular comorbidity. Exp Dermatol (2011) 20:303–7. 10.1111/j.1600-0625.2011.01261.x 21410760

[16] Gisondi P Fantin F Del Giglio M Valbusa F Marino F Zamboni M Chronic plaque psoriasis is associated with increased arterial stiffness. Dermatology (2009) 218:110–3. 10.1159/000182256 19060461

[17] Fang N Jiang M Fan Y. Association between psoriasis and subclinical atherosclerosis: a meta-analysis. Medicine (Baltimore) (2016) 95:e3576. 10.1097/MD.0000000000003576 27196459 PMC4902401

[18] Flammer AJ Ruschitzka F Psoriasis and atherosclerosis: two plaques, one syndrome? Eur Heart J (2012) 33:1989–91. 10.1093/eurheartj/ehr425 22108835

[19] Davidovici BB Sattar N Prinz J Puig L Emery P Barker JN Psoriasis and systemic inflammatory diseases: potential mechanistic links between skin disease and co-morbid conditions. J Invest Dermatol (2010) 130:1785–96. 10.1038/jid.2010.103 20445552

[20] Ryan C Kirby B. Psoriasis is a systemic disease with multiple cardiovascular and metabolic comorbidities. Dermatol Clin (2015) 33:41–55. 10.1016/j.det.2014.09.004 25412782

[21] Ghazizadeh R Shimizu H Tosa M Ghazizadeh M. Pathogenic mechanisms shared between psoriasis and cardiovascular disease. Int J Med Sci (2010) 7:284–9. 10.7150/ijms.7.284 20827428 PMC2934727

[22] Chen S Crother TR Arditi M. Emerging role of IL-17 in atherosclerosis. J Innate Immun (2010) 2:325–33. 10.1159/000314626 20505315 PMC2895754

[23] Mehta NN Li K Szapary P Krueger J Brodmerkel C. Modulation of cardiometabolic pathways in skin and serum from patients with psoriasis. J Transl Med (2013) 11:194. 10.1186/1479-5876-11-194 23965158 PMC3765699

[24] Kolliker Frers RA Cosentino V Tau J Kerzberg EM Urdapilleta A Chiocconi M Immune-mediated inflammation promotes subclinical atherosclerosis in recent-onset psoriatic arthritis patients without conventional cardiovascular risk factors. Front Immunol (2018) 9:139. 10.3389/fimmu.2018.00139 29535705 PMC5834432

[25] Libby P Ridker PM Hansson GK Leducq Transatlantic Network on Atherothrombosis Inflammation in atherosclerosis: from pathophysiology to practice. J Am Coll Cardiol (2009) 54:2129–38. 10.1016/j.jacc.2009.09.009 19942084 PMC2834169

[26] Prodanovich S Ma F Taylor JR Pezon C Fasihi T Kirsner RS. Methotrexate reduces incidence of vascular diseases in veterans with psoriasis or rheumatoid arthritis. J Am Acad Dermatol (2005) 52:262–7. 10.1016/j.jaad.2004.06.017 15692471

[27] Micha R Imamura F Wyler von Ballmoos M Solomon DH Hernán MA Ridker PM Systematic review and meta-analysis of methotrexate use and risk of cardiovascular disease. Am J Cardiol (2011) 108:1362–70. 10.1016/j.amjcard.2011.06.054 21855836 PMC3196048

[28] Wu JJ Poon KY Channual JC Shen AY. Association between tumor necrosis factor inhibitor therapy and myocardial infarction risk in patients with psoriasis. Arch Dermatol (2012) 148:1244–50. 10.1001/archdermatol.2012.2502 22911151

[29] Ahlehoff O Skov L Gislason G Lindhardsen J Kristensen SL Iversen L Cardiovascular disease event rates in patients with severe psoriasis treated with systemic anti-inflammatory drugs: a Danish real-world cohort study. J Intern Med (2013) 273:197–204. 10.1111/j.1365-2796.2012.02593.x 22963528

[30] Hu SC Lan CE. Psoriasis and cardiovascular comorbidities: focusing on severe vascular events, cardiovascular risk factors and implications for treatment. Int J Mol Sci (2017) 18:E2211. 10.3390/ijms18102211 29065479 PMC5666891

[31] Mottaghi A Ebrahimof S Angoorani P Saboor-Yaraghi AA. Vitamin A supplementation reduces IL-17 and RORc gene expression in atherosclerotic patients. Scand J Immunol (2014) 80:151–7. 10.1111/sji.12190 24845870

[32] Mahmoudi MJ Saboor-Yaraghi AA Zabetian-Targhi F Siassi F Zarnani AH Eshraghian MR Vitamin A decreases cytotoxicity of oxidized low-density lipoprotein in patients with atherosclerosis. Immunol Invest (2016) 45:52–62. 10.3109/08820139.2015.1095208 26700065

[33] Boehncke S Fichtlscherer S Salgo R Garbaraviciene J Beschmann H Diehl S Systemic therapy of plaque-type psoriasis ameliorates endothelial cell function: results of a prospective longitudinal pilot trial. Arch Dermatol Res (2011) 303:381–8. 10.1007/s00403-010-1108-6 21170539

[34] Puig L Cardiovascular risk and psoriasis: the role of biologic therapy. Actas Dermosifiliogr (2012) 103:853–62. 10.1016/j.ad.2012.02.003 23157913

[35] Rungapiromnan W Yiu ZZN Warren RB Griffiths CEM Ashcroft DM. Impact of biologic therapies on risk of major adverse cardiovascular events in patients with psoriasis: systematic review and meta-analysis of randomized controlled trials. Br J Dermatol (2017) 176:890–901. 10.1111/bjd.14964 27518205 PMC5412670

[36] Raimondo A Balato A Megna M Balato N Limitations of current monoclonal antibodies for plaque-type psoriasis and an outlook for the future. Expert Opin Biol Ther (2018) 18(6):605–7. 10.1080/14712598.2018.1479738 29788767

[37] Balato A Mattii M Caiazzo G Raimondo A Patruno C Balato N IL-36γ is involved in psoriasis and allergic contact dermatitis. J Invest Dermatol (2016) 136:1520–3. 10.1016/j.jid.2016.03.020 27021407

[38] Schiattarella M Caiazzo G Di Caprio R Lembo S Raimondo A Ayala F Paraoxonases and psoriasis: negative imbalance of anti-oxidant endogenous mechanisms. G Ital Dermatol Venereol (2017). 10.23736/S0392-0488.17.05537-7 28509526

[39] Furue M Tsuji G Chiba T Kadono T. Cardiovascular and metabolic diseases comorbid with psoriasis: beyond the skin. Intern Med (2017) 56:1613–9. 10.2169/internalmedicine.56.8209 28674347 PMC5519460

[40] Karczewski J Dobrowolska A Rychlewska-Hańczewska A Adamski Z. New insights into the role of T cells in pathogenesis of psoriasis and psoriatic arthritis. Autoimmunity (2016) 49:435–50. 10.3109/08916934.2016.1166214 27050731

[41] Baliwag J Barnes DH Johnston A. Cytokines in psoriasis. Cytokine (2015) 73:342–50. 10.1016/j.cyto.2014.12.014 25585875 PMC4437803

[42] Testa M Yeh M Lee P Fanelli R Loperfido F Berman JW Circulating levels of cytokines and their endogenous modulators in patients with mild to severe congestive heart failure due to coronary artery disease or hypertension. J Am Coll Cardiol (1996) 28:964–71. 10.1016/S0735-1097(96)00268-9 8837575

[43] Karbach S Croxford AL Oelze M Schüler R Minwegen D Wegner J Interleukin 17 drives vascular inflammation, endothelial dysfunction, and arterial hypertension in psoriasis-like skin disease. Arterioscler Thromb Vasc Biol (2014) 34:2658–68. 10.1161/ATVBAHA.114.304108 25341795

[44] Bozkurt B Kribbs SB Clubb FJ Jr Michael LH Didenko VV Hornsby PJ Pathophysiologically relevant concentrations of tumor necrosis factor-α promote progressive left ventricular dysfunction and remodeling in rats. Circulation (1997) 97:1382–91. 10.1161/01.CIR.97.14.1382 9577950

[45] Kubota T McTiernan CF Frye CS Slawson SE Lemster BH Koretsky AP Dilated cardiomyopathy in transgenic mice with cardiac-specific overexpression of tumor necrosis factor-α. Circ Res (1997) 81:627–35. 10.1161/01.RES.81.4.627 9314845

[46] Cesari M Penninx BW Newman AB Kritchevsky SB Nicklas BJ Sutton-Tyrrell K Inflammatory markers and cardiovascular disease: the health, aging and body composition [health ABC] study. Am J Cardiol (2003) 92:522–8. 10.1016/S0002-9149(03)00718-5 12943870

[47] Balato A Scala E Balato N Caiazzo G Di Caprio R Monfrecola G Biologics that inhibit the Th17 pathway and related cytokines to treat inflammatory disorders. Expert Opin Biol Ther (2017) 17:1363–74. 10.1080/14712598.2017.1363884 28791896

[48] FDA Approves Biosimilar Products. Silver Spring, MD: US Food and Drug Administration (2018). Available from: https://www.fda.gov/Drugs/Development Approval Process/How Drugsare Developed and Approved/Approval Applications/Therapeutic Biologic Applications/Biosimilars/ucm580432.htm (Accessed: May 29, 2018).

[49] Balato A Schiattarella M Di Caprio R Lembo S Mattii M Balato N Effects of adalimumab therapy in adult subjects with moderate-to-severe psoriasis on Th17 pathway. J Eur Acad Dermatol Venereol (2014) 28:1016–24. 10.1111/jdv.12240 24033358

[50] Caldarola G De Simone C Carbone A Tulli A Amerio P Feliciani C TNF and its receptors in psoriatic skin, before and after treatment with etanercept. Int J Immunopathol Pharmacol (2009) 22:961–6. 10.1177/039463200902200411 20074459

[51] Cordiali-Fei P Trento E D’Agosto G Bordignon V Mussi A Ardigò M Decreased levels of metalloproteinase-9 and angiogenic factors in skin lesions of patients with psoriatic arthritis after therapy with anti-TNF-α. J Autoimmune Dis (2006) 5:3–5. 10.1186/1740-2557-3-5 PMC1601955 17022813

[52] Strober B Foley P Philipp S Zhang N Kaur P Evaluation of efficacy and safety of ABP 501 in a phase 3 study in subjects with moderate to severe plaque psoriasis: 52-week results. J Am Acad Dermatol (2016) 74(5, Suppl 1):AB249 10.1016/j.jaad.2016.02.972

[53] Momenta Pharmaceuticals Announces Positive Top-Line Phase 3 Results for M923, a Proposed Humira (Adalimumab) Biosimilar [News Release]. Cambridge, MA: Momenta Pharmaceuticals, Inc (2016). Available from: http://ir.momentapharma.com/releasedetail.cfm?ReleaseID=1001255 (Accessed: January 25, 2017).

[54] Kellen R Goldenberg G. Biosimilars in psoriasis: the future or not? Cutis (2017) 99(2):116–20. 28319617

[55] Campanati A Orciani M Ganzetti G Consales V Di Primio R Offidani A. The effect of etanercept on vascular endothelial growth factor production by cutaneous mesenchymal stem cells from patients with psoriasis. J Int Med Res (2016) 44:6–9. 10.1177/0300060515593229 27683131 PMC5536541

[56] Henning M Göcker Tekin H Skov L Egeberg A Effects of biologic therapy on cardiovascular disease in psoriasis. Curr Derm Rep (2018) 7:37–42. 10.1007/s13671-018-0210-4

[57] Gkalpakiotis S Arenbergerova M Gkalpakioti P Potockova J Arenberger P Kraml P. Impact of adalimumab treatment on cardiovascular risk biomarkers in psoriasis: results of a pilot study. J Dermatol (2017) 44:363–9. 10.1111/1346-8138.13661 27774694

[58] Goldminz AM Suárez-Fariñas M Wang AC Dumont N Krueger JG Gottlieb AB. CCL20 and IL22 messenger RNA expression after adalimumab vs methotrexate treatment of psoriasis: a randomized clinical trial. JAMA Dermatol (2015) 151:837–46. 10.1001/jamadermatol.2015.0452 25946554 PMC5788701

[59] Boehncke S Salgo R Garbaraviciene J Beschmann H Hardt K Diehl S Effective continuous systemic therapy of severe plaque-type psoriasis is accompanied by amelioration of biomarkers of cardiovascular risk: results of a prospective longitudinal observational study. J Eur Acad Dermatol Venereol (2011) 25:1187–93. 10.1111/j.1468-3083.2010.03947.x 21241371

[60] Marra M Campanati A Testa R Sirolla C Bonfigli AR Franceschi C Effect of etanercept on insulin sensitivity in nine patients with psoriasis. Int J Immunopathol Pharmacol (2007) 20:731–6. 10.1177/039463200702000408 18179745

[61] Pina T Armesto S Lopez-Mejias R Genre F Ubilla B Gonzalez-Lopez MA Anti-TNF-α therapy improves insulin sensitivity in non-diabetic patients with psoriasis: a 6-month prospective study. J Eur Acad Dermatol Venereol (2015) 29:1325–30. 10.1111/jdv.12814 25353352

[62] Pina T Genre F Lopez-Mejias R Armesto S Ubilla B Mijares V Anti-TNF-a therapy reduces retinol-binding protein 4 serum levels in non-diabetic patients with psoriasis: a 6-month prospective study. J Eur Acad Dermatol Venereol (2015) 30:92–5. 10.1111/jdv.13005 25650695

[63] Jókai H Szakonyi J Kontár O Marschalkó M Szalai K Kárpáti S Impact of effective tumor necrosis factor-alfa inhibitor treatment on arterial intima-media thickness in psoriasis: results of a pilot study. J Am Acad Dermatol (2013) 69:523–9. 10.1016/j.jaad.2013.06.019 23891393

[64] Bissonnette R Tardif JC Harel F Pressacco J Bolduc C Guertin MC Effects of the tumor necrosis factor-α antagonist adalimumab on arterial inflammation assessed by positron emission tomography in patients with psoriasis: results of a randomized controlled trial. Circ Cardiovasc Imaging (2013) 6:83–90. 10.1161/CIRCIMAGING.112.975730 23204039

[65] Hjuler KF Bøttcher M Vestergaard C Bøtker HE Iversen L Kragballe K. Association between changes in coronary artery disease progression and treatment with biologic agents for severe psoriasis. JAMA Dermatol (2016) 152:1114–21. 10.1001/jamadermatol.2016.1984 27385305

[66] Ahlehoff O Hansen PR Gislason GH Frydland M Bryld LE Elming H Myocardial function and effects of biologic therapy in patients with severe psoriasis: a prospective echocardiographic study. J Eur Acad Dermatol Venereol (2016) 30:819–23. 10.1111/jdv.13152 25845841

[67] Herédi E Végh J Pogacsas L Gaspar K Varga J Kincse G Subclinical cardiovascular disease and it’s improvement after longterm TNF-alpha inhibitor therapy in severe psoriatic patients. J Eur Acad Dermatol Venereol (2016) 30:1531–6. 10.1111/jdv.13649 27393182

[68] Wu JJ Poon KY Association of ethnicity, tumor necrosis factor inhibitor therapy, and myocardial infarction risk in patients with psoriasis. J Am Acad Dermatol (2013) 69(1):167–8. 10.1016/j.jaad.2013.02.019 23768295

[69] Deswal A Bozkurt B Seta Y Parilti-Eiswirth S Hayes FA Blosch C Safety and efficacy of a soluble P75 tumor necrosis factor receptor (Enbrel, etanercept) in patients with advanced heart failure. Circulation (1999) 99:3224–6. 10.1161/01.CIR.99.25.3224 10385494

[70] Mann DL McMurray JJ Packer M Swedberg K Borer JS Colucci WS Targeted anticytokine therapy in patients with chronic heart failure: results of the Randomized Etanercept Worldwide Evaluation (RENEWAL). Circulation (2004) 109:1594–602. 10.1161/01.CIR.0000124490.27666.B2 15023878

[71] Chung ES Packer M Lo KH Fasanmade AA Willerson JT Anti-TNF Therapy Against Congestive Heart Failure Investigators Randomized, double-blind, placebo-controlled, pilot trial of infliximab, a chimeric monoclonal antibody to tumor necrosis factor-alpha, in patients with moderate-to-severe heart failure: results of the anti-TNF therapy against congestive heart failure (ATTACH) trial. Circulation (2003) 107:3133–40. 12796126 10.1161/01.CIR.0000077913.60364.D2

[72] Pham T Bachelez H Berthelot JM Blacher J Bouhnik Y Claudepierre P TNF alpha antagonist therapy and safety monitoring. Joint Bone Spine (2011) 78(Suppl 1):15–185. 10.1016/S1297-319X(11)70001-X 21703545

[73] AbbVie. Safety and Efficacy Study of Adalimumab in the Treatment of Plaque Psoriasis. (2017). Available from: https://clinicaltrials.gov/show/NCT01646073 (Accessed: January 17, 2017).

[74] Gottlieb AB Matheson RT Lowe N Krueger GG Kang S Goffe BS A randomized trial of etanercept as monotherapy for psoriasis. Arch Dermatol (2003) 139:1627–32. 10.1001/archderm.139.12.1627 14676082

[75] Bachelez H van de Kerkhof PC Strohal R Kubanov A Valenzuela F Lee JH Tofacitinib versus etanercept or placebo in moderate-to-severe chronic plaque psoriasis: a phase 3 randomised non-inferiority trial. Lancet (2015) 386:552–61. 10.1016/S0140-6736(14)62113-9 26051365

[76] Griffiths CE Reich K Lebwohl M van de Kerkhof P Paul C Menter A Comparison of ixekizumab with etanercept or placebo in moderate-to-severe psoriasis (UNCOVER-2 and UNCOVER-3): results from two phase 3 randomised trials. Lancet (2015) 386:541–51. 10.1016/S0140-6736(15)60125-8 26072109

[77] Solomon DH Massarotti E Garg R Liu J Canning C Schneeweiss S Association between disease-modifying antirheumatic drugs and diabetes risk in patients with rheumatoid arthritis and psoriasis. JAMA (2011) 305:2525–31. 10.1001/jama.2011.878 21693740

[78] Abuabara K Lee H Kimball AB. The effect of systemic psoriasis therapies on the incidence of myocardial infarction: a cohort study. Br J Dermatol (2011) 165:1066–73. 10.1111/j.1365-2133.2011.10525.x 21777216

[79] Wu JJ Guérin A Sundaram M Dea K Cloutier M Mulani P Cardiovascular event risk assessment in psoriasis patients treated with tumor necrosis factor-α inhibitors versus methotrexate. J Am Acad Dermatol (2017) 76:81–90. 10.1016/j.jaad.2016.07.042 27894789

[80] Gisondi P Cotena C Tessari G Girolomoni G. Anti-tumour necrosis factor-alpha therapy increases body weight in patients with chronic plaque psoriasis: a retrospective cohort study. J Eur Acad Dermatol Venereol (2008) 22:341–4. 10.1111/j.1468-3083.2007.02429.x 18005022

[81] Cawthorn WP Sethi JK TNF-α and adipocyte biology. FEBS Lett (2007) 582:117–31. 10.1016/j.febslet.2007.11.051 18037376 PMC4304634

[82] Reich K Langley RG Papp KA Ortonne JP Unnebrink K Kaul M A 52-week trial comparing briakinumab with methotrexate in patients with psoriasis. N Engl J Med (2011) 365:1586–96. 10.1056/NEJMoa1010858 22029980

[83] Reich K Langley RG Lebwohl M Szapary P Guzzo C Yeilding N Cardiovascular safety of ustekinumab in patients with moderate to severe psoriasis: results of integrated analyses of data from phase II and III clinical studies. Br J Dermatol (2011) 164:862–72. 10.1111/j.1365-2133.2011.10257.x 21332467

[84] Tzellos T Kyrgidis A Zouboulis CC Re-evaluation of the risk for major adverse cardiovascular events in patients treated with anti-IL-12/23 biological agents for chronic plaque psoriasis: a meta-analysis of randomized controlled trials. J Eur Acad Dermatol Venereol (2013) 27:622–7. 10.1111/j.1468-3083.2012.04500.x 22404103

[85] Langley R Valdes J Gordon K Papp K P3312. Long-term safety and efficacy of ABT-874 for the treatment of moderate to severe psoriasis: interim analysis from an open-label extension study. J Am Acad Dermatol (2011) 64:AB148.

[86] Tzellos T Kyrgidis A Trigoni A Zouboulis CC Point: major adverse cardiovascular events and anti-IL 12/23 agents. J Am Acad Dermatol (2014) 70(2):380–1. 10.1016/j.jaad.2013.07.050 24438954

[87] van de Kerkhof PC Griffiths CE Reich K Leonardi CL Blauvelt A Tsai TF Secukinumab long-term safety experience: a pooled analysis of 10 phase II and III clinical studies in patients with moderate to severe plaque psoriasis. J Am Acad Dermatol (2016) 75:83–98.e4. 10.1016/j.jaad.2016.03.024 27180926

[88] Egeberg A Ottosen MB Gniadecki R Broesby-Olsen S Dam TN Bryld LE Safety, efficacy and drug survival of biologics and biosimilars for moderate-to-severe plaque psoriasis. Br J Dermatol (2018) 178:509–19. 10.1111/bjd.16102 29094341

[89] Strober B Leonardi C Papp KA Mrowietz U Ohtsuki M Bissonnette R Short- and long-term safety outcomes with ixekizumab from 7 clinical trials in psoriasis: etanercept comparisons and integrated data. J Am Acad Dermatol (2017) 76:432–40. 10.1016/j.jaad.2016.09.026 27889292

[90] Papp KA Reich K Paul C Blauvelt A Baran W Bolduc C A prospective phase III, randomized, double-blind, placebo-controlled study of brodalumab in patients with moderate-to-severe plaque psoriasis. Br J Dermatol (2016) 175:273–86. 10.1111/bjd.14493 26914406

[91] Luger D Silver PB Tang J Cua D Chen Z Iwakura Y Either a Th17 or a Th1 effector response can drive autoimmunity: conditions of disease induction affect dominant effector category. J Exp Med (2008) 205:799–810. 10.1084/jem.20071258 18391061 PMC2292220

[92] Mitra A Fallen RS Lima HC. Cytokine-based therapy in psoriasis. Clin Rev Allergy Immunol (2013) 44:173–82. 10.1007/s12016-012-8306-2 22426927

[93] Lee E Trepicchio WL Oestreicher JL Pittman D Wang F Chamian F Increased expression of interleukin 23 p19 and p40 in lesional skin of patients with psoriasis vulgaris. J Exp Med (2004) 199:125–30. 10.1084/jem.20030451 14707118 PMC1887731

[94] Chan JR Blumenschein W Murphy E Diveu C Wiekowski M Abbondanzo S IL-23 stimulates epidermal hyperplasia via TNF and IL-20R2-dependent mechanisms with implications for psoriasis pathogenesis. J Exp Med (2006) 203:2577–87. 10.1084/jem.20060244 17074928 PMC2118145

[95] Correia LCL Andrade BB Borges VM Clarêncio J Bittencourt AP Freitas R Prognostic value of cytokines and chemokines in addition to the GRACE score in non-ST-elevation acute coronary syndromes. Clin Chim Acta (2010) 411:540–5. 10.1016/j.cca.2010.01.011 20083097

[96] Abbas A Gregersen I Holm S Daissormont I Bjerkeli V Krohg-Sørensen K Interleukin 23 levels are increased in carotid atherosclerosis. Stroke (2015) 46:793–9. 10.1161/STROKEAHA.114.006516 25649806

[97] Uyemura LL Demer SC Castle D Jullien JA Berliner MK Gately RR Cross-regulatory roles of interleukin (IL)-12 and IL-10 in atherosclerosis. J Clin Invest (1996) 97:2130–8. 10.1172/JCI118650 8621803 PMC507288

[98] Yong K Dogra G Boudville N Chan D Adams L Ching H Interleukin-12 is associated with arterial stiffness in healthy individuals. Am J Hypertens (2013) 26:159–62. 10.1093/ajh/hps032 23382399

[99] Papp KA Langley RG Lebwohl M Krueger GG Szapary P Yeilding N Efficacy and safety of ustekinumab, a human interleukin-12/23 monoclonal antibody, in patients with psoriasis: 52-week results from a randomised, double-blind, placebo-controlled trial (PHOENIX 2). Lancet (2008) 371:1675–84. 10.1016/S0140-6736(08)60726-6 18486740

[100] Leonardi CL Kimball AB Papp KA Yeilding N Guzzo C Wang Y Efficacy and safety of ustekinumab, a human interleukin-12/23 monoclonal antibody, in patients with psoriasis: 76-week results from a randomised, double-blind, placebo-controlled trial (PHOENIX 1). Lancet (2008) 371:1665–74. 10.1016/S0140-6736(08)60725-4 18486739

[101] Gordon KB Langley RG Gottlieb AB Papp KA Krueger GG Strober BE A phase III, randomized, controlled trial of the fully human IL-12/23 mAb briakinumab in moderate-to-severe psoriasis. J Invest Dermatol (2012) 132:304–14. 10.1038/jid.2011.304 22011907

[102] Gottlieb AB Leonardi C Kerdel F Mehlis S Olds M Williams DA. Efficacy and safety of briakinumab vs. etanercept and placebo in patients with moderate to severe chronic plaque psoriasis. Br J Dermatol (2011) 165:652–60. 10.1111/j.1365-2133.2011.10418.x 21574983

[103] Strober BE Crowley JJ Yamauchi PS Olds M Williams DA. Efficacy and safety results from a phase III, randomized controlled trial comparing the safety and efficacy of briakinumab with etanercept and placebo in patients with moderate to severe chronic plaque psoriasis. Br J Dermatol (2011) 165:661–8. 10.1111/j.1365-2133.2011.10419.x 21574984

[104] Ryan C Leonardi CL Krueger JG Kimball AB Strober BE Gordon KB Association between biologic therapies for chronic plaque psoriasis and cardiovascular events: a meta-analysis of randomized controlled trials. JAMA (2011) 306:864–71. 10.1001/jama.2011.1211 21862748

[105] Bradburn MJ Deeks JJ Berlin JA Russell Localio A. Much ado about nothing: a comparison of the performance of meta-analytical methods with rare events. Stat Med (2007) 26:53–77. 10.1002/sim.2528 16596572

[106] Dommasch ED Troxel AB Gelfand JM Major cardiovascular events associated with anti-IL 12/23 agents: a tale of two meta-analyses. J Am Acad Dermatol (2013) 68:863–5. 10.1016/j.jaad.2013.01.011 23602173

[107] Deeks JJ. Issues in the selection of a summary statistic for meta-analysis of clinical trials with binary outcomes. Stat Med (2002) 21:1575–600. 10.1002/sim.1188 12111921

[108] Tzellos T Kyrgidis A Toulis K Biologic therapies for chronic plaque psoriasis and cardiovascular events. JAMA (2011) 306:2095 10.1001/jama.2011.1660 22089716

[109] Kimball AB Papp KA Wasfi Y Chan D Bissonnette R Sofen H Long-term efficacy of ustekinumab in patients with moderate-to-severe psoriasis treated for up to 5 years in the PHOENIX 1 study. J Eur Acad Dermatol Venereol (2012) 27:1535–45. 10.1111/jdv.12046 23279003

[110] Gottlieb A Menter A Mendelsohn A Shen Y-K Li S Guzzo C Ustekinumab, a human interleukin 12/23 monoclonal antibody, for psoriatic arthritis: randomised, double-blind, placebo-controlled, crossover trial. Lancet (2009) 373:633–40. 10.1016/S0140-6736(09)60140-9 19217154

[111] Sandborn WJ Feagan BG Fedorak RN Scherl E Fleisher MR Katz S A randomized trial of ustekinumab, a human interleukin-12/23 monoclonal antibody, in patients with moderate-to-severe Crohn’s disease. Gastroenterology (2008) 135:1130–41. 10.1053/j.gastro.2008.07.014 18706417

[112] Sandborn WJ Gasink C Gao L-L Blank MA Johanns J Guzzo C Ustekinumab induction and maintenance therapy in refractory Crohn’s disease. N Engl J Med (2012) 367:1519–28. 10.1056/NEJMoa1203572 23075178

[113] Callen JP Are Major Adverse Cardiovascular Events Associated With Anti–IL-12/23 Therapies? (2017). Available from: http://www.jwatch.org/jd201109160000001/2011/09/16/are-major-adverse-cardiovascular-events (Accessed: January 17, 2017).

[114] Reddy M Torres G McCormick T Marano C Cooper K Yeilding N Positive treatment effects of ustekinumab in psoriasis: analysis of lesional and systemic parameters. J Dermatol (2010) 37:413–25. 10.1111/j.1346-8138.2010.00802.x 20536646

[115] Taleb S Tedgui A Mallat Z. IL-17 and Th17 cells in atherosclerosis: subtle and contextual roles. Arterioscler Thromb Vasc Biol (2015) 35:258–64. 10.1161/ATVBAHA.114.303567 25234818

[116] Takatori H Kanno Y Watford WT Tato CM Weiss G Ivanov II Lymphoid tissue inducer-like cells are an innate source of IL-17 and IL-22. J Exp Med (2009) 206:35–41. 10.1084/jem.20072713 19114665 PMC2626689

[117] Kagami S Rizzo HL Lee JJ Koguchi Y Blauvelt A. Circulating Th17, Th22, and Th1 cells are increased in psoriasis. J Invest Dermatol (2010) 130:1373–83. 10.1038/jid.2009.399 20032993 PMC2892169

[118] Wu D Hou SY Zhao S Hou LX Jiao T Xu NN Efficacy and safety of interleukin-17 antagonists in patients with plaque psoriasis: a meta-analysis from phase 3 randomized controlled trials. J Eur Acad Dermatol Venereol (2017) 31:992–1003. 10.1111/jdv.14125 28107570

[119] Erbel C Dengler TJ Wangler S Lasitschka F Bea F Wambsganss N Expression of IL-17A in human atherosclerotic lesions is associated with increased inflammation and plaque vulnerability. Basic Res Cardiol (2011) 106:125–34. 10.1007/s00395-010-0135-y 21116822

[120] Taleb S Romain M Ramkhelawon B Uyttenhove C Pasterkamp G Herbin O Loss of SOCS3 expression in T cells reveals a regulatory role for interleukin-17 in atherosclerosis. J Exp Med (2009) 206:2067–77. 10.1084/jem.20090545 19737863 PMC2757872

[121] Hashmi S Zeng QT. Role of interleukin-17 and interleukin-17-induced cytokines interleukin-6 and interleukin-8 in unstable coronary artery disease. Coron Artery Dis (2006) 17:699–706. 10.1097/01.mca.0000236288.94553.b4 17119379

[122] Cheng X Yu X Ding YJ Fu QQ Xie JJ Tang TT The Th17/Treg imbalance in patients with acute coronary syndrome. Clin Immunol (2008) 127:89–97. 10.1016/j.clim.2008.01.009 18294918

[123] Eid RE Rao DA Zhou J Lo SF Ranjbaran H Gallo A Interleukin-17 and interferon-gamma are produced concomitantly by human coronary artery-infiltrating T cells and act synergistically on vascular smooth muscle cells. Circulation (2009) 119:1424–32. 10.1161/CIRCULATIONAHA.108.827618 19255340 PMC2898514

[124] Simon T Taleb S Danchin N Laurans L Rousseau B Cattan S Circulating levels of interleukin-17 and cardiovascular outcomes in patients with acute myocardial infarction. Eur Heart J (2013) 34:570–7. 10.1093/eurheartj/ehs263 22956509

[125] Golden JB McCormick TS Ward NL. IL-17 in psoriasis: implications for therapy and cardiovascular co-morbidities. Cytokine (2013) 62:195–201. 10.1016/j.cyto.2013.03.013 23562549 PMC3640599

[126] Gordon KB Blauvelt A Papp KA Langley RG Luger T Ohtsuki M Phase 3 trials of ixekizumab in moderate-to-severe plaque psoriasis. N Engl J Med (2016) 375:345–56. 10.1056/NEJMoa1512711 27299809

[127] Lebwohl M Strober B Menter A Gordon K Weglowska J Puig L Phase 3 studies comparing brodalumab with ustekinumab in psoriasis. N Engl J Med (2015) 373:1318–28. 10.1056/NEJMoa1503824 26422722

